# Expressions of Topo IIα and Ki67 in breast cancer and its clinicopathologic features and prognosis

**DOI:** 10.12669/pjms.35.3.81

**Published:** 2019

**Authors:** Guangyu Sun, Shuyan Wang, Ying Wang

**Affiliations:** 1 *Guangyu Sun, Oncology Department I, Binzhou People’s Hospital, Shandong, 256600, China*; 2 *Shuyan Wang Oncology Department II, Binzhou People’s Hospital, Shandong, 256600, China*; 3 *Ying Wang Oncology Department I, Binzhou People’s Hospital, Shandong, 256600, China*

**Keywords:** Breast cancer, Ki67, Topo IIα

## Abstract

**Objective:**

Breast cancer is one of the most common malignant tumors in women, and its incidence in women has ranked the first place among all malignant cancers. The evolution of breast cancer which is highly heterogeneous is a complex biological process involving multiple factors, genes and stages. Therefore, studying the expression and interaction of related genes is of great significance to further revealing of the mechanism of the occurrence and development of breast cancer and reduction of its resistance to chemotherapy. The purpose of this study was to investigate expressions of Ki67 and Topoisomerase IIα (Topo IIα) in breast cancer tissues and to explore the relationship between expressions of Ki67 and Topo IIα and the pathological characteristics and prognosis of breast cancer.

**Methods:**

Tissues with pathological changes and peripheral normal breast tissues were collected from 66 patients who underwent breast cancer treatment. Ki67 and Topo IIα in the breast cancer tissues and normal breast tissues were detected using immunohistochemical method. The relationships of Ki67 and Topo IIα with general clinicopathologic features and prognosis were analyzed.

**Results:**

It was found that the positive expressions of Ki67 and Topo IIα in the breast cancer tissues were higher than those in the normal tissues; the positive expression rate of Ki67 was in a correlation with diameter of breast cancer and lymphatic metastasis (P<0.05); the positive expression rate of Topo IIα was in a correlation with clinical stage, diameter of cancer and lymphatic metastasis (P<0.05). Multi-factor logistic regression analysis suggested that lymphatic metastasis and clinical stage were the independent predictive factors for positive expression of Topo IIα and lymphatic metastasis was the independent predictive factor for positive expression of Ki-67. Breast cancer patients with negative Ki67 and Topo IIα had significantly higher local recurrence rate than those who had positive Ki67 and Topo IIα (P<0.05) after surgery. The median time to progression (TTP) of the patients with positive expression of Topo IIα was lower than that with negative expression (P<0.05). The median TTP of the patients with negative and positive expression of Ki67 had no significant difference (P>0.05).

**Conclusion:**

Topo IIα and Ki67 can be used for reflecting the proliferation activity of cancer cells and can affect the postoperative recurrence and survival time after surgery. They may be involved in the occurrence, development and metastasis of breast cancer. They are expected to be indicators in the prognosis prediction of breast cancer and potential treatment targets.

## INTRODUCTION

Breast cancer is one of the most common malignant tumors in women all over the world. The average annual incidence of breast cancer is about 30/100,000-50/100,000, and it is increasingly constantly. It tends to occur to younger women and has seriously affected women’s physical and mental health.^1^,^2^ A survey shows that the incidence of breast cancer in developed countries in Europe and the United States is as high as 80/ten thousand.^3^ There is no nationwide data on the incidence of breast cancer in China, but the predicted data show that there will be more than 100 cases of breast cancer among ten thousand women by 2021.^4^ A large number of studies have confirmed that mutations in key genes controlling cell growth play a leading role in the occurrence and development of breast cancer and the expression and level of some cell molecules can reflect the differentiation degree of tumor cells.^5^,^6^ With the rapid development of molecular biology, more and more attentions have been paid to the study of tumor markers such as Ki-67 and Topoisomerase II α (Topo IIα) in breast cancer.

Ki67 protein is located in nucleus and its coding gene is located on chromosome 10. It is believed that Ki67 is closely related to cell mitosis. It is not expressed in stationary cells, but only exists in proliferative nuclei. It is an important indicator of cell proliferation activity.^7^ A large number of studies have found that the expression of Ki67 in malignant tumors is closely related to pathological grade,^8^,^9^ clinical efficacy and prognosis, and has been widely used in the detection of proliferation activity of various tumors, as well as the evaluation of tumor invasion and prognosis. Topo IIα is the coding gene of a DNA topoisomerase. A foreign study found that Topo IIα as a predictive indicator of efficacy of anthracycline-based chemotherapy and recurrence and metastasis of tumor has high application values in patients with breast cancer especially triple-negative breast cancer and can guide the selection of treatment scheme.^10^ In this experiment, expressions of Ki67 and Topo IIα in breast cancer tissues were studied using immunohistochemical method, and their relationships with pathological characteristics and prognosis were analyzed, aiming to provide a theoretical basis for the evaluation of clinical prognosis of breast cancer and a new idea for further exploration of individual treatment.

## METHODS

Tissues with pathological changes and peripheral normal breast tissues were collected from sixty-six patients who received breast cancer surgery in our hospital between June 2013 and December 2017. Patients included were females who were pathologically diagnosed as breast cancer, did not undergo chemo radiotherapy before surgery and had no cancer cells in pathological examination of normal breast tissues. They aged 31~83 years (average 38.28±12.36 years). There were 11 cases of TNM stage I, 27 cases of stage II, 23 cases of stage III and 5 cases of stage IV; 30 of them had lymphatic metastasis, while 36 patients had no lymphatic metastasis. As to histopathological type, there were 33 cases of invasive ductal carcinoma, 20 cases of invasive lobular carcinoma, 7 cases of mucinous adenocarcinoma and 6 cases of intraductal carcinoma. Ethical approval has been obtained from the ethical committee of our hospital, and all the patients signed informed consent.

The tissue samples were fixed using 10% formaldehyde, dehydrated, embedded, sliced, dewaxed and hydrated after being resected from human body. The structure of tissues was observed using hematoxylin-eosin staining. Expressions of Ki67 and Topo IIα in breast cancer and normal breast tissues were detected using immunohistochemical method. Phosphate buffer solution (PBS) was taken as the negative control instead of primary antibody. Rabbit anti-human Topo IIα monoclonal antibody and rabbit anti-human Ki-67 monoclonal antibody (Fujian Maixin Biotechnology Co., Ltd., China) were used.

It was determined as negative if Topo II nucleus was stained and the percentage of stained cells was lower than 10% and as positive if higher than 10%. It was determined as positive if Ki67 nucleus was stained, negative if the percentage of positive cells was lower than 5% and positive if the percentage of positive cells was lower than 6%. Evaluation criteria followed the scoring method of Mahler-Araujo et al.: 0 point for the percentage of positive cells lower than 10%,^11^ 1 point for the percentage of positive cells between 10% and 25%, 2 points for the percentage of positive cells between 25% and 50%, 3 points for the percentage of positive cells between 50% and 75%, and 4 points for the percentage of positive cells higher than 75%; 0~3 points indicated negative expression and > 3 points indicated positive expression.

The patients were followed up through phone call and output treatment. The prognosis of the patients was tracked and recorded to understand whether the disease condition was stable and whether there was local recurrence or distant metastasis and specific death causes. The follow up started from the day of surgery and ended up on December 2017.

All the data of this study were statistically analyzed and processed using SPSS ver. 21.0. Categorical data were expressed as n or percentage and processed using Chi-square test. Fisher’s exact test was used when the theoretical frequency of samples was smaller than 1; indicators with statistical significance were given multi-factor logistic regression analysis to screen independent predictive indicators. The relationship between expressions of Ki67 and Topo IIα and time to progression (TTP) was analyzed using Kaplan-Meier survival analysis method; survival curve was drawn. The TTP of patients with different expressions of Ki67 and Topo IIα was compared using Log-rank test. α=0.05 was taken as the significant level, and P<0.05 meant difference had statistical significance.

## RESULTS

**Fig.1:**
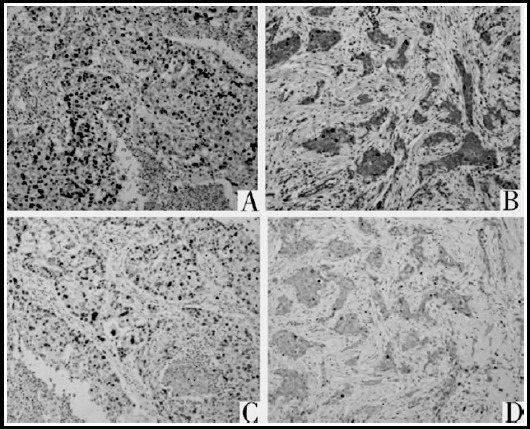
Expression of Topo IIα and Ki67 (immunohistochemical, ×100) (A: positive expression of Topo IIα, B: negative expression of Topo IIα; C: positive expression of Ki67; D: negative expression of Ki67).

The positive expression rate of Ki67 and Topo IIα in breast cancer tissues was 80.3% and 69.7% respectively (Fig.1), and both of them had no positive expression in normal breast tissues; the difference between the two groups was statistically significant (P<0.05, Table-I).

**Table-I:** Expressions of Ki67 and Topo IIα in breast cancer and normal breast tissues [n(%)].

Tissues	Ki67	Topo IIα
Breast cancer tissues	53(80.3)	46(69.7)
Normal breast tissues	0(0)	0(0)
X^2^	52.859	66.672
P	<0.05	<0.05

In breast cancer tissues, the positive rate of Ki67 was in a correlation with diameter of lesions and lymphatic metastasis (P<0.05). The positive rate of Topo IIα was in a correlation with clinical stage and diameter of breast cancer and lymphatic metastasis (P<0.05, Table-II).

**Table-II:** The relationship between expressions of Topo Iiα and Ki67 and clinicopathological parameters.

Factor		n	Topo IIα			Ki67		

			Number of positive cases	X^2^	P value	Number of positive cases	X^2^	P value
Age (year)	< 50	36	20	0.273	>0.05	22	1.024	>0.05
	≥50	30	19			24		
Menstrual status	Non-menopause	43	22	0.317	>0.05	40	1.648	>0.05
	Menopausal	23	11			20		
Diameter of lesion (cm)	<2	35	18	8.146	<0.05	19	7.969	<0.05
	≥2	31	28			27		
Lymphatic metastasis	With	30	13	9.723	<0.05	16	11.174	<0.05
	Without	36	28			32		
Pathological pattern	Invasive ductal carcinoma	33	21	1.318	>0.05	24	1.324	>0.05
	Intraductal carcinoma	6	4			4		
	Invasive lobular carcinoma	20	10			12		
	Mucinous adenocarcinoma	7	4			5		
Clinical stage	Stage I and II	38	19	4.576	<0.05	14	2.178	>0.05
	Stage III and IV	28	21			22		

Multi-factor logistic regression analysis was performed on diameter of lesion, lymphatic metastasis and clinical stage which were found having statistical significance in the single-factor analysis. The results demonstrated that lymphatic metastasis and clinical stage were the independent predictive factors of positive expression of Topo IIα and lymphatic metastasis is the independent predictive factor of positive expression of Ki-67 (Table-III).

**Table-III:** The results of the multi-factor regression analysis of Topo IIα and Ki67 in breast cancer tissues.

Factor	Topo IIα			Ki-67		

	P value	OR value	95% CI	P value	OR value	95% CI
Diameter of lesion	0.738	0.864	0.386~1.919	0.172	0.508	0.192~1.353
Lymphatic metastasis	0.008	1.779	1.171~2.695	0.032	1.721	1.029~2.835
Clinical stage	0.000	0.125	0.037~0.401	0.745	0.854	0.331~2.200

***Note:*** OR: odd ratio.

During the follow-up period, 28 out of 66 breast cancer patients had local recurrence. All patients were followed up for 49.5 months, the shortest 12 months and the longest 72 months. The local recurrence rates of breast cancer patients with positive expressions of Ki67 and Topo IIα were 47.2% (25/53) and 50.0% (23/46) respectively, which were significantly higher than those of breast cancer patients with negative expressions (23.1% (3/13) and 25.0% (5/20)). The difference was statistically significant (X^2^=5.132, P<0.05; X^2^=5.358, P<0.05).

**Fig.2:**
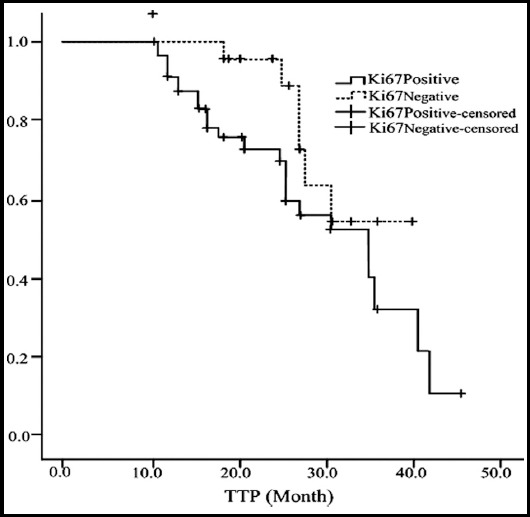
Effects of expression of Ki67 of breast cancer patients on TTP.

**Fig.3:**
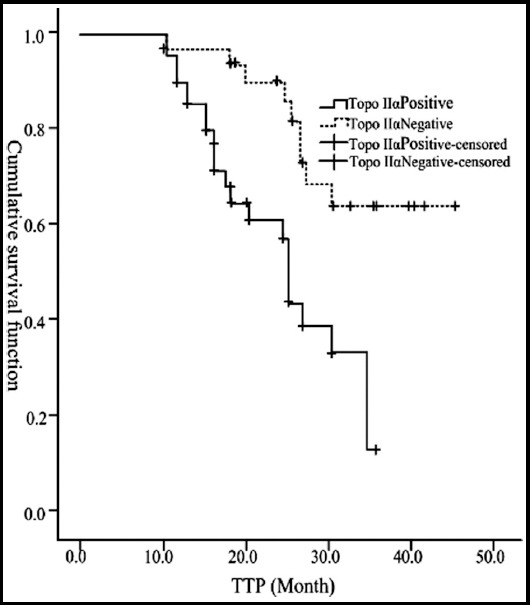
Effects of expression of Topo IIα of breast cancer patients on TTP.

The median TTP was 34.7 months in Ki67-positive patients (95% CI: 26.2~43.4) and 36.6 months in Ki67-negative patients (95% CI: 27.7-45.6). Log-rank test suggested that there was no significant difference in the median TTP between Ki67-negative and Ki67-positive patients (X^2^=2.775, P>0.05, Fig.2). The median TTP was 25.4 months in Topo IIα-positive patients (95% CI: 24.2~26.4) and 41.3 months in Topo IIα-negative patients (95% CI: 39.1~43.4). Log-rank test suggested that the median TTP of Topo II-negative patients were significantly different from that of Topo IIα-positive patients (X^2^=12.168, P<0.05, Fig.3).

## DISCUSSION

With the continuous progress of early diagnosis and surgery, surgery combined with postoperative radiotherapy and chemotherapy has become the most effective treatment for breast cancer patients, which can prolong the survival time of patients. For patients with late pathological stage and patients who cannot undergo radical resection or had incomplete section, local recurrence or metastasis can occur in a short term after operation,^12^,^13^ In addition, the insensitivity of some patients to chemotherapy and the failure of chemotherapy caused by multi-drug resistance have been a major problem in the field of cancer. Therefore, exploring the evaluation index of chemotherapy effect and prognosis of breast cancer has become an important research topic. With the rapid development of molecular biology, more and more genes have been labeled to reflect the existence of tumor cells.^14^ Similarly, some key genes controlling the growth of breast cells have been gradually recognized. These key genes play an important role in the expression of some molecules in cells and the expression level, thus forming a macroscopic manifestation of differentiation degree of tumor cells.

Ki67 is an antigen that exists in the nuclear matrix of proliferating cells. It is located on chromosome 10 of human body and is considered an important marker for evaluating the proliferative activity of tumor cells in recent years.^15^ In normal tissues, the expression of Ki67 is generally lower than 300, and increased in cancerous tissues. The expression level increases gradually with the development of the disease. The results of this study showed that the positive expression rate of Ki67 in breast cancer tissues was significantly higher than that in adjacent tissues and the positive rate of Ki67 was related to tumor diameter and lymphatic metastasis of breast cancer (P<0.05), and the multi-factor logistic regression analysis suggested that lymphatic metastasis was the independent predictive indicator of positive expression of Ki67, which was consistent with most previous studies,^16^,^17^ It suggested that the positive expression of Ki67 was closely related to the growth, invasion and lymphatic metastasis of breast cancer and that the proliferation of breast cancer cells was active, Ki67-positive tumor cell grew fast and had high invasion and poor prognosis.

Topo IIα mainly distributes in proliferating cells and has a specific effect on cell cycle. It mainly affects phase S and G2/M. Topo IIα can give a sound respond to cell proliferation and is a major prognostic indicator of malignant tumor.^18^ Park et al. found that Topo IIα was closely related to the prognosis of malignant tumors and it was different in many cancers and was usually overexpressed.^19^ A study has shown that anthracycline chemotherapeutics was more effective than TopoIIα when TopolIα expression was positive in patients with primary breast cancer.^20^ The present study found that the positive expression rate of Topo IIα was 69.7% in breast cancer tissues and 0% in normal adjacent tissues (P<0.05) and the positive rate of Topo IIα was also different in breast cancer lesions with different clinical stages and size, with or without lymphatic metastasis (P<0.05), suggesting that Topo IIα was also closely related to biological behaviors of breast cancer such as stage and growth. The single-factor and multi-factor logistic regression analysis indicated that lymphatic metastasis was the independent predictive factor of positive expression of Topo IIα. Lymphatic metastasis is an important factor which is used for evaluating the prognosis of invasive breast cancer conventionally; the severer the lymphatic metastasis, the later the stage of breast cancer and the poorer the prognosis.^21^ Therefore it is assumed that TopoIIα as the important indicator that can reflect the proliferative activity of cancer cells was in a correlation with the malignant progress, metastasis and prognosis of breast cancer

In addition, the survival analysis showed that the median TTP of Topo IIα-positive patients was significantly lower than that of Topo IIα-negative patients, and the difference was statistically significant (P<0.05). It indicated that the expression of Topo IIα could be an independent prognostic factor for breast cancer, and overexpression of Topo IIα usually indicated poor prognosis. The possible reason was that the overexpression of Topo IIα could induce lower differentiation and higher invasiveness of breast cancer by inhibiting the function of tumor suppressor genes and enhancing the proliferation activity of tumor cells, which shortened the survival time of patients. It was similar to the results of Knoop et al.^22^ Knoop et al. found that the disease-free survival and overall survival time of breast cancer patients with negative expression of Topo IIα were longer, and their 3-year survival rate was significantly higher than patients with positive expression.

## CONCLUSION

In conclusion, Ki67 and Topo IIα may together participate in the occurrence, development and metastasis of breast cancer and joint detection of Topo IIα and Ki67 can provide a better reference for the prediction of the nature, chemotherapeutic efficacy and prognosis of breast cancer. But this study is retrospective and single-center; hence the results might be biased. Retrospective, multi-center randomized controlled trials which include more samples are needed for further verification.

### Authors’ Contribution

**GYS:** Study design, data collection and analysis.

**GYS, SYW & YW:** Manuscript preparation, drafting and revising.

**SGY:** Review and final approval of manuscript.
